# Coping with Abiotic Stress in Plants—An Endomembrane Trafficking Perspective

**DOI:** 10.3390/plants11030338

**Published:** 2022-01-27

**Authors:** Miguel Sampaio, João Neves, Tatiana Cardoso, José Pissarra, Susana Pereira, Cláudia Pereira

**Affiliations:** 1 GreenUPorto—Sustainable Agrifood Production Research Centre/Inov4Agro, Department of Biology, Faculty of Sciences, University of Porto, Rua do Campo Alegre, s/nº, 4169-007 Porto, Portugal; miguelfsampaio@hotmail.com (M.S.); jpissarr@fc.up.pt (J.P.); 2 Department of Biology, Faculty of Sciences, University of Porto, Rua do Campo Alegre, s/nº, 4169-007 Porto, Portugal; jpneves99@hotmail.com (J.N.); tatiffcardoso_902@hotmail.com (T.C.)

**Keywords:** endomembrane system, vacuolar trafficking, stress, vacuole, endoplasmic reticulum, Golgi apparatus, unconventional routes

## Abstract

Plant cells face many changes through their life cycle and develop several mechanisms to cope with adversity. Stress caused by environmental factors is turning out to be more and more relevant as the human population grows and plant cultures start to fail. As eukaryotes, plant cells must coordinate several processes occurring between compartments and combine different pathways for protein transport to several cellular locations. Conventionally, these pathways begin at the ER, or endoplasmic reticulum, move through the Golgi and deliver cargo to the vacuole or to the plasma membrane. However, when under stress, protein trafficking in plants is compromised, usually leading to changes in the endomembrane system that may include protein transport through unconventional routes and alteration of morphology, activity and content of key organelles, as the ER and the vacuole. Such events provide the tools for cells to adapt and overcome the challenges brought on by stress. With this review, we gathered fragmented information on the subject, highlighting how such changes are processed within the endomembrane system and how it responds to an ever-changing environment. Even though the available data on this subject are still sparse, novel information is starting to untangle the complexity and dynamics of protein transport routes and their role in maintaining cell homeostasis under harsh conditions.

## 1. Introduction

Climate changes stand, nowadays, as the foremost threat to human and environmental health, causing crop failures worldwide and leading to food safety issues [1]. As sessile organisms, plants evolved the ability to adapt to, and take advantage of, changes in climate and environment [2]. The diverse environmental stresses often activate signals and pathways involved in similar cellular responses: overexpression of antioxidants, accumulation of solutes, changes in protein trafficking and endomembrane remodelling [3,4,5,6]. In recent years, by using high throughput screening techniques, such as microarrays and RNA sequencing, it was possible to identify many stress-related genes. These techniques provide us with important information and suggest genes among which it is possible to identify new markers for assisted selection of crop varieties resistant to stress. Nevertheless, changes in the transcriptome are still the result of a complex series of events, and our understanding of the mechanisms of stress response is only partial. One of the most relevant mechanisms occurs at the endomembrane level, in particular in what concerns inter-organellar communications [7,8]. The identification of the specific roles of each player in the game turns out to be an important factor for the genetic improvement of plants, because the positive adaptation probably depends on synergistic effects and balanced interactions among proteins that are normally not related [9]. Recent experimental evidence [10] suggests various classes of proteins (such as aquaporins, soluble N-ethylmaleimide-sensitive factor attachment protein receptors (SNAREs), ATPase pumps or channels) to control specific events of membrane transport, leading to important events of cell reorganization under adverse environmental conditions. Several research groups found interesting connections between stress tolerance and membrane rearrangements not observed before, as is the case for a potassium channel—AKT1/KC1 (a shaker-like potassium channel)—selectively accumulated on small vacuoles [11] and sufficient to confer stress tolerance when overexpressed. Nonetheless, the connection between membranous structure architecture and stress tolerance is not sufficiently investigated. In fact, the idea that endomembrane trafficking is tightly linked to stress signalling pathways has been backed up by a number of studies, unfortunately, without a deeper understanding of the underlying mechanisms. Over the past decades, remarkable progress has been made in understanding the mechanisms related to protein sorting, and special emphasis has been given to the study of proteins targeted to the vacuolar pathways and the sorting mechanisms involved due to their importance in plant cell homeostasis. However, more recent data suggest that the classical view of protein transport to the vacuole might be challenged by alternative routes that have begun to be described in more recent years [12,13,14]. These alternative routes are thought to be one of the plant responses to adverse conditions. As such, it is thought that the vacuolar trafficking pathways might be altered under specific conditions to better accomplish the plant’s needs. In addition, the cell cytoskeleton also plays an important role in the response/adaptation to stress, since the interaction between the actin cytoskeleton and the endomembrane system is crucial to preserve many aspects of plant cell function and development [15]. In this review, we intend to bring together the mechanistic information available on the endomembrane system’s adaptations and responses to stress, focusing on the vacuolar route and associated vesicle transport mechanisms.

## 2. The ER as a Cellular Stress Sensor

The Endoplasmic Reticulum (ER), being a network of tubules and cisternae that spreads along the entire cell and connects with several other organelles, plays a major role in maintaining cellular homeostasis and in perceiving and spreading external signals [7]. The ER is the organelle that mediates the stress response in animals and in plants [16,17,18]. Abiotic stress can be responsible for the misfolding of proteins and their accumulation, causing an ER stress situation [19,20,21]. In response, some mechanisms are activated by the cell in order to maintain the ER’s homeostasis, for example, the expression of genes encoding chaperones and other proteins with protein folding capacity, degradation associated with the ER or the cutback of protein translation loaded to the ER [19,22] (Table 1). One of these mechanisms is the binding of the unfolded proteins to BIP proteins (binding proteins), which will activate bZIP transcription factors, such as bZIP17/bZIP28, that are transported to the Golgi to be cleaved [18,19]. This transport will upregulate genes involved in the ER stress pathway in order to restore ER homeostasis [18]. Furthermore, this transport may mediate the overexpression of genes involved in stress response as the bZIP28 leading to the activation of heat stress response genes [18,23] and bZIP17 leading to the activation of salt stress responses [20,24]. In this way, it is possible to observe that, in heat and salt stress situations, ER stress responses are often activated [18,23,24]. However, there are other important proteins involved in the unfolded protein responses (UPR) that respond to adverse environmental conditions, such as inositol-requiring enzyme-1 (IRE1), an ER resident transmembrane protein [18,25]. This protein is described to be involved in the heat stress response. When it is activated by heat, IRE1 splices bZIP60 mRNA, which is required for the activation of genes involved in the ER’s stress reaction [26]. This protein also regulates the stress transcriptome by degrading several mRNAs [27,28]. In addition to the mechanism previously described, other UPR are activated in this type of stress as the activation of BiP; however, its overexpression is not relevant, meaning that salt stress can simply promote the misfolding of a different group of proteins [18,24,29,30]. Recently, another transcriptional factor modulating plant UPR has been identified. The nonexpressor of PR1 genes 1 (NPR1), a crucial redox-regulated master regulator of salicylic acid (SA)-dependent responses to pathogens, has been demonstrated to suppress the transcriptional role of bZIP28 and bZIP60 in ER stress responses. Upon ER stress-induced reduction of the cytosolic redox potential, NPR1 is translocated to the nucleus and physically interacts with bZIP28 and bZIP60, acting as an antagonist of such UPR factors to optimize their cytoprotective role in the UPR. NPR1 functions in plant UPR-monitoring may promote a negative feedback loop that is important in balancing energy consumption and maintaining basal cellular homeostasis during ER stress signalling [31]. In addition to cell-intrinsic UPR signalling, a non-cell autonomous component has been demonstrated to be regulated by the intercellular movement of bZIP60 facilitating systemic UPR signalling. Evidences show that the sbZIP60 protein is able to traffic across cells and induces the activity of the promoter of a target gene, efficiently stimulating UPR gene expression in cells distally from the site where ER stress occurs. Such findings suggest that ER stress systemic signalling may constitute a mechanism of anticipation to a potentially-upcoming ER stress, as the cells of yet-unchallenged tissues are prepared by inducing the accumulation of transcripts of ER stress attenuating proteins [32].

Alongside ER stress responses, other mechanisms of compartmentalization involving the ER are activated under stress, including autophagy. Macroautophagy consists of a conserved cellular process that involves the sequestration of cytosolic components by a newly formed, double membrane vesicle, termed an autophagosome, that is eventually directed to the plant vacuole [33,34]. Notably, selective autophagy was also reported to act in special trafficking routes, delivering vacuolar resident proteins to function in this organelle. This role of autophagy in the biogenesis-mediating process is in contrast to the degradative nature classically associated with it. It was shown that inducing ER stress in Arabidopsis results in the delivery of ER components, such as the ER membrane decorated with ribosomes, to vacuoles via autophagy [34], further strengthening the possible involvement of autophagy in storage protein trafficking. In addition, it is now recognized that following ER stress, ER components are delivered for degradation via autophagy in both yeast and mammalian cells [35,36,37]. In this aspect, plants are not different from other eukaryotes, and accumulation of Atg8-positive bodies that co-localized with the ER marker GFP-HDEL was detected following ER stress. The presence of autophagic bodies containing ER membrane was also detected by electron-microscopy in vacuoles of ER-stressed plants [34]. Another study performed by Bao and collaborators [38] showed that IRE1b is connected with this type of response. However, this pathway is not related to the BZIP60 but to the regulated IRE1-dependent decay of messenger RNA (RIDD), in which mRNAs of gene encoding factors that inhibit the activation of autophagy processes are degraded by IRE1 upon ER stress [39], such as BGLU21, a member of the β-glucosidase family that is one of the principal components of the ER bodies.

Being the starting point of the endomembrane system, the ER is in a privileged position to recognize extracellular cues and coordinate the cellular response to adverse and challenging conditions to the cell. Its nature as a central network that extends throughout the cell facilitates several contact points with other organelles, representing the high complexity of the ER mechanisms essential to maintaining the function of cellular homeostasis and signalling cascades.

## 3. The Vacuole as a Key Organelle in Stress Response

Vacuoles can occupy up to 80% of the cell volume, serve physical and metabolic functions and are essential in cellular responses to general cell homeostasis, as well as abiotic and biotic factors [40,41]. These organelles usually store water, nutrients, ions and secondary metabolites, but can also be a deposit site for toxic cell residues, waste products and excess solutes [42,43,44,45] and are involved in programmed cell death [46]. In plant cells, there are two main types of vacuoles: the protein storage vacuole (PSV) and the lytic vacuole (LV). Typically, proteins accumulate in the PSVs due to their higher pH and lower hydrolytic activity, when compared to the LVs, and they predominate in storage tissues (as cotyledons, endosperm, tubers) and vegetative tissues of adult plants (bark, leaves, pods) [47,48]. In contrast, LVs are mostly found in vegetative tissues and are used for storage and deposit of unwanted products. Due to the LVs’ acidic pH and high hydrolytic activity, this type of vacuole modulates the degradation of a panoply of macromolecules and other compounds [49,50]. Initially, it was not expected to find both types of vacuoles in the same cell, but studies performed in root tip cells of barley and pea seedlings showed otherwise [51,52]. Additionally, a study conducted using the model plant *Arabidopsis thaliana* reported that, during its germination, the LV is embedded in the PSV and then derives from it, instead of being generated de novo [53]. The existence of two different types of vacuoles implies that plants have distinct trafficking pathways and mechanisms for different proteins. Additionally, the presence of LVs and PSVs in the same cell might work as a plant flexibility mechanism that is hypothesized to relate with changing environmental conditions [54,55,56,57].

A recent publication by Neves and collaborators has highlighted that Arabidopsis plants under abiotic stress show a differential expression of genes related to vacuolar trafficking, with an enhancement of the route to the PSV [6]. In fact, plants under abiotic stress are able to modulate their development and growth by altering morphological and cellular mechanisms, and cells’ responses/adaptations to stress might involve changes in the distribution and sorting of specific proteins and molecules. Several studies also show the important role of the vacuole as a defence mechanism against abiotic stress. Indeed, the vacuole seems to react to stress through multiple mechanisms, such as toxic product accumulation and cell-turgor pressure maintenance. A study using suspension-cultured cells of mangrove (*Bruguiera sexangula*) shows the rapid increase in vacuolar volume when cells are submitted to salt stress, at the expense of decreasing cytoplasm volume, to maintain turgor pressure, probably through the increase in Na^+^ concentration in the vacuole [58]. Another study using *Arabidopsis thaliana* shows the importance of the vacuole when the plant is under oxidative stress. In fact, the vacuole accumulated high levels of GSSG (oxidized glutathione) as a mechanism to protect the cell from an excessively positive shift in cytosolic glutathione redox potential [59]. The vacuole is also involved in mechanisms to fight environmental stress, such as reducing high ion levels toxicity in the cytoplasm to avoid cell death. A study by Tang et al. [60] uncovered a novel function of the Calcineurin B-like (CLB) interacting protein kinases’ (CIPK) (CBL–CIPK) signalling network in excessive Mg2^+^ vacuolar sequestration to help plants thrive under Mg2^+^ stress. The described Mg2^+^ partitioning process in the vacuole controlled by the CBL–CIPK pathway may represent a general mechanism underlying the detoxification of other ions, including Na^+^ (Table 2).

Conversely to abiotic stress, where the integrity of the vacuole is extremely important for the homeostasis of the cell, during pathogen infections the vacuole disruption and the release of its contents have to occur (for a review on the subject, see [61]). As an innate mechanism defence, the vacuole accumulates large amounts of hydrolytic enzymes, such as proteases and antimicrobial compounds that are eventually released under pathogen attack in a process that it is not completely understood [62,63]. Two different mechanisms associated with programmed cell death (PCD) and hypersensitive response have been described for the release of vacuolar contents [64]. Briefly, one involves vacuolar processing enzymes (VPEs) and the disruption of the tonoplast and the other implicates the fusion between the tonoplast and the plasma membrane (PM) (Table 2). In both cases, the result is the release of vacuolar contents.

As critical as changes in vacuolar trafficking, alterations in vacuolar morphology are a key feature of cell homeostasis under stress and also contribute to plant homeostasis. These adaptations are tightly connected with the actin cytoskeleton and regulated by SNARE proteins, allowing a structural reorganization of the vacuolar network while maintaining its dynamics [65,66].

## 4. Vacuolar Transport—A Cellular Response to Stress

The protein trafficking to the vacuole is a fine-tuned communication system mediated by vesicles and different types of receptors. This allows the existence of a differential sorting process of proteins, leading to a different destination, depending on the receptors and vesicles used [67,68]. The vacuolar sorting receptors (VSRs) are involved in the transport of soluble cargoes by the conventional pathway, being responsible for the binding and release of cargo and also the control of the trafficking from and to the prevacuolar compartment (PVC) [14,69]. Besides these receptors, receptor homology region-transmembrane domain-RING-H2 (RMR) proteins have been identified as part of the traffic to the PSV. However, these types of receptors cannot be recycled back [67,70,71]. Another distinctive factor for the final destination of the vacuolar proteins is the type of vesicles. Clathrin coated vesicles (CCVs) are involved in the post-Golgi transport, being localized at the *trans* Golgi Network (TGN), and are responsible for the trafficking of proteins to the LV [67,70,72]. In contrast to the CCVs, dense vesicles (DVs) are larger carriers that fuse with PVCs and travel to the PSV [14,73,74,75]. Taken together, given all the data available on protein trafficking to the vacuole, gathered over many years of research, it is clear that it is a flexible and highly coordinated network [14,76]. As such, it is not surprising that, under abiotic stress, this tight balance can be altered to face the cell’s needs and, ultimately, the plant, in order to adapt and to be able to prosper.

The alterations in the vacuolar trafficking as a cellular response to stress have not been characterized yet, and only a few studies approach this theme (Table 3). Nevertheless, some isolated observations and reports are worth mentioning, as they may open the door for more focused research. In a recent study, Neves and collaborators [6] evaluated how different abiotic stresses affect the endomembrane system in *A. thaliana* by studying the expression of several endomembrane system effectors. The authors show that *AtRMR1, AtVSR1, AtSYP51* and *AtVTI12* genes, involved in the PSV sorting, are positively regulated under abiotic stress, while genes involved in the LV sorting, such as *AtVTI11* and *AtVSR2,* are downregulated. These findings enable the authors to create a hypothesis where the PSV route would be enhanced under abiotic stress conditions, in detriment of the LV pathway. Despite being very preliminary, this study points to several important genes in the vacuolar route that may be useful to fully understand how the cell copes with adverse conditions. One example is the v-SNAREs *VTI12* and its homologue *VTI11*, which function in different vesicle transport pathways, mediating the transport to different vacuolar types [77]. *VTI12*, however, has broader roles, participating in the docking and fusion of autophagic vesicles [78]. It is also part of a protein complex, together with SYP61 and SYP41, localized at the TGN. SYP61 has been implicated in osmotic stress responses [79], and it is thought that it may also be involved in stress-responsive transport mechanisms, similar to what has been described for SYP121 at the plasma membrane [10]. Being in a complex with SYP61, it is possible that *VTI12* may also participate in this mechanism. In fact, It has been shown that, in Arabidopsis plants grown under abiotic stress, *VTI12* expression is 20–30-fold higher than in control conditions [6], which is indicative of a putative role in cells’ adaptation or response to stress. Furthermore, the VSR’s implicated in the trafficking to the PSV also seem to respond to stress. Recently, Wang and collaborators [80] proposed a novel role for AtVSR1 in osmotic stress tolerance and in the regulation of abscisic acid (ABA) biosynthesis, which is an important regulator of the signalling pathways induced by osmotic stresses. The authors used a *vsr* mutant and showed that the vacuolar trafficking mediated by *VSR1* was necessary for a response, in terms of ABA biosynthesis and to attain osmotic stress tolerance. In another study, Arabidopsis plants overexpressing *AtRabG3e* showed increased tolerance to salt and osmotic stress, along with a reduction in the accumulation of reactive oxygen species [81]. The Rab GTPases consist of a large family of proteins with a role in regulating vesicle targeting and specificity [82], and *AtRabG3e* participates in membrane fusion between the PVC and the vacuole, reinforcing the role of this pathway in adaptations to stress. Apart from the conventional route, the endocytic route to the vacuole has also been implicated in plant salt stress tolerance. This was shown in a work by Leshem and collaborators [83], where the suppression of the v-SNARE *AtVAMP7C*, essential for endosomal vesicle fusion with the tonoplast, had a positive impact in improving plant salt tolerance by inhibiting the fusion of H_2_O_2_-containing vesicles with the vacuole.

As a whole, protein trafficking to the vacuole plays an important role both in responses and adaptations to stress, with the SNARE proteins being fundamental in the process (See [84] for a review on SNAREs in plant stress responses). Additionally, the involvement of other post-Golgi pathways should be explored, as in the case of Adaptor protein 3 (AP-3) and the adaptor complex that interacts with *VTI12* in the TGN [85]. This route seems to influence plant responses to stress conditions interacting in parallel with the conventional pathway and allowing a faster delivery of essential proteins for the biogenesis of the vacuole. Additionally, DVs-mediated transport is also a good alternative to the conventional transport under these adverse conditions and one that still requires investigation.

## 5. Unconventional Vacuolar Routes, or a Way to Get to the Vacuole Faster

In the past years, several studies have characterized proteins and vacuolar signals that do not follow the conventional sorting route. Some alternative sorting pathways, such as AP-3 and dense vesicles sorting, require the Golgi apparatus, but others also appear to be Golgi-independent [14]. Very little is known about the relationship between unconventional sorting routes and stress, but these alternative routes might be activated under stress to better match the plant’s specific needs at the cellular level (Table 4). In fact, direct ER-to-vacuole pathways appear to be linked to autophagy-related processes, which can be caused by multiple types of environmental disturbances. In recent years, several proteins or vacuolar sorting determinants have been described to follow an alternative, ER-to-vacuole, route [86,87,88]. Among them, cardosin A Plant Specific Insert (PSI) is an interesting case, as other related domains do not have this ability [13]. It is thought that other undescribed unconventional routes similar to the PSI-mediated vacuolar sorting act when plants face stress situations, providing the option to sort proteins through the conventional pathway or through a direct ER-to-vacuole transport. In fact, a recent study published in *Conference Proceedings* [89] showed that overexpression of PSIB in *Arabidopsis thaliana* correlates with salt and osmotic stress conditions, in some cases improving plant fitness. A different type of proteins that also appear to relate with salt stress are cysteine proteases that accumulate in long ER bodies, both in seedlings (as seen in *Vigna mungo* [90] and *Ricinus communis* [91]) or vegetative tissues’ epidermis (*Arabidopsis thaliana* [92]) that eventually fuse with the vacuole. These proteins, along with vacuolar processing enzymes, are responsible for the degradation of storage proteins during plant development, but observations that ER bodies’ direct fusion with the vacuole may be triggered by stress [92] enable a new perspective on the importance of this type of transport. A similar mechanism has been described upon pathogen attacks, when ER bodies filled with defence proteins such as pathogenesis-related 1 (PR1) or plant defensin 1.2 (PDF1.2) are formed (for review, see [61]) and eventually fuse with the plasma membrane or with the vacuole in a Golgi-independent manner. Furthermore, autophagic compartments are frequently induced by stress [5,93], and autophagy markers are frequently observed in the ER and vacuole membranes [94]. However, it is still not clear how autophagy per se can contribute to vacuolar sorting and other mechanisms or regulators should be involved. Anthocyanins imported to the vacuole during cycles of stress/starvation provide an interesting example of unconventional trafficking, in this case involving the exocyst pathway and its role in autophagy and defence in plants [95,96].

After looking at all the examples available, it is crucial to study the direct ER-to-vacuole transport in plants under stress. In fact, characterizing unconventional sorting routes together with stress responses would provide new insights into the little knowledge available so far. The Golgi bypass might play a strong role in stress responses, as it makes protein transport to the vacuole faster and more dynamic. In fact, several unconventional routes are not constitutive but induced by changes in the cell environment.

## 6. A Role for the Cytoskeleton in Keeping Cell Homeostasis under Stress

The cytoskeleton concept has been changed from a static supportive structure to a dynamic process in energetic equilibrium that adapts its functions to driving changes and stress responses in a fine-tune time and space resolution [97]. Myosin motors along with actin filament bundles predominantly drive intracellular transport in plant cells. Changes in the rate of actin remodelling also affect its functionality, as observed by alteration in Golgi body motility [98]. Both remodelling of the ER and Golgi movement are inhibited by depolymerization of actin, demonstrating the importance of the actin cytoskeleton [99,100]. Mutant knock-out analysis of four members of the Myosin XI family (xi-k, xi-1, xi2 and xi-i) demonstrate that these proteins are important for normal whole-organism and cellular growth as well as Golgi body dynamics [101]. However, microtubules are thought to be essential during critical stages of plant cell development [102]. Considering that stress is a condition quite challenging to the cell, the hypothesis that the cytoskeleton network also has to adapt needs to be tested, since the interaction with membranes is critical for the self-organization of the cell. In a review of the complexity of organelle movement within the plant secretory pathway, Brandizzi and Wasteneys [102] argue that the actin-centric view of the motility of secretory organelles has been challenged by recent advances and revisited reports that support the relevant role of microtubules in plant cell development, positioning of Golgi stacks, involvement in cellulose synthesis and auxin polar transport. A milestone in the elucidation of the connection between endomembrane trafficking and microtubules was the work of Ambrose and collaborators [103] that, using hybrid and in vivo bimolecular fluorescence complementation techniques, discovered that microtubule-associated protein CLASP interacts with the retromer, facilitating the association between TGN/early endosomes and cortical microtubules via an interaction with sorting nexin1 (SNX1). SNX1 is a component of the retromer protein complex responsible for recycling the plasma membrane auxin efflux carrier PIN2, and thus controlling auxin transport.

Reinforcing the role of microtubules in organelle positioning and function, further studies described the tethering of compartments carrying cellulose synthase complexes to microtubules. Contrarily to some cell wall polysaccharides synthesized by glycosyl transferases and modified by enzymes located in the Golgi [102], cellulose is produced from multi-enzyme complexes at the plasma membrane. This multi-enzyme complex must be delivered to the appropriate places at the PM. Cellulose synthase (CESA) complexes (CSCs) are assembled in the Golgi and secreted to the plasma membrane through the *trans* Golgi network (TGN) compartment [104]. Studies in intracellular trafficking of cellulose synthase complexes identified microtubule-associated cellulose synthase compartments, namely small CesA-containing compartments (SmaCCs) [105] and microtubule-associated cellulose synthase compartments (MASCs) [106]. Osmotic stress or inhibition of cellulose synthesis induces endocytosis of the cellulose synthase complex, where organelles containing CESA accumulate and interact strongly with cortical microtubules [105,106] (Table 5). After relieving osmotic stress, these organelles deliver CSC to the PM [105], leading to the hypothesis that some of these microtubule-associated compartments are functional secretory vesicles in cellular stress [104] and that the SmaCCs associated with CSC delivery may represent a specialized secretory pathway involved in cell wall biosynthesis. The SmaCC/MASC-mediated fast recovery of CSCs upon stress relief is dependent on CELLULOSE SYNTHASE INTERACTIVE1 (CSI1), a protein involved in the link between CSCs and cortical microtubules [107]. Additionally, AP2M, a component in clathrin-mediated endocytosis, plays a role in the formation of SmaCCs/MASCs. Lei and collaborators [107] propose a model in which, in the process of endocytosis, CSI1-dependent SmaCCs/MASCs are formed, allowing a quick regulation of cellulose synthesis under abiotic stress (Table 5). All these approaches contribute to unravelling a spatiotemporal model of trafficking events in cell wall deposition in normal and stressful conditions.

The NET super-family of actin binding proteins [15] interact directly with F-actin and are recruited to different membrane compartments via a C-terminal sequence. Several of such proteins have been identified so far and each label a specific compartment: NET1A—plasma membrane, NET4A—tonoplast, and NET3B—endoplasmic reticulum. Moreover, there is evidence that some endoplasmic reticulum–plasma membrane contact site (EPCSs) proteins associated with actin, a group that includes NET1A, react to extracellular signals such as stress related to pathogen infection [108] (Table 5). Protein complexes consisting of the actin-binding protein NET3C (At2g47920) and the membrane-anchored protein VAP27 (At3g60600), which has an affinity for microtubules, are proposed to specify the contact sites between the cortical endoplasmic reticulum network and the plasma membrane [109].

In summary, organizing the endomembrane trafficking depends on the fine-tune regulation of such transport in space and time involving both actin and microtubule-based mechanisms. Moreover, transport of cargo molecules between compartments occurs mainly by vesicle shuttles (i.e., transport vesicles), where the cytoskeleton has a role in facilitating vesicle transport from one compartment to another [110]. The relevance of this “shuttle transport” might attain more significant outlines in a context of cellular reorganization when the plant is facing adverse conditions.

## 7. Conclusions and Future Directions

**Figure 1:**
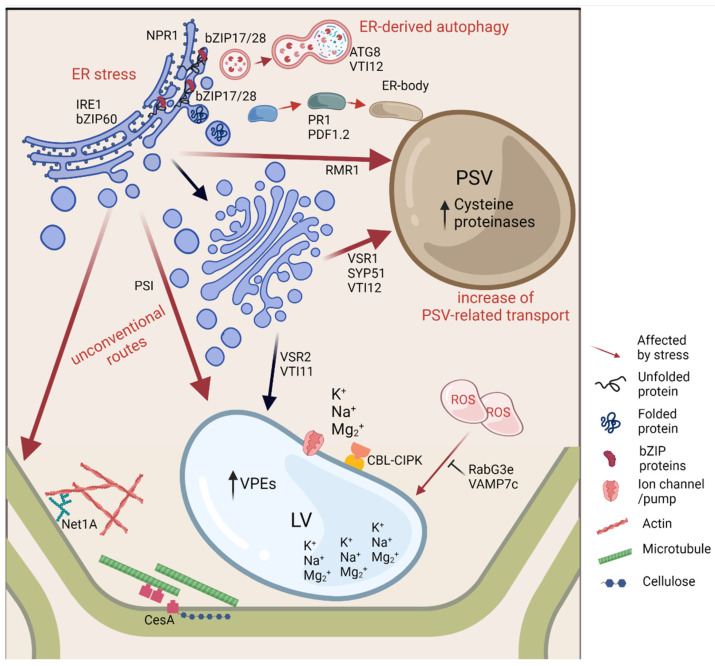
Schematic representation of the alterations observed in endomembrane trafficking and associated protein effectors during abiotic stress conditions. When facing adverse environmental conditions, plant cells’ response involves the de novo expression of several genes and proteins and an accumulation of reactive oxygen species (ROS) in several organelles or in the cytoplasm. The endoplasmic reticulum is affected by stress by inducing an ER stress response (bZIP17/28, NPR1, IRE1, bZIP60), activating genes responsible for the ER-unfolded protein response and inducing ER-derived autophagy (ATG8, *VTI12*). Unconventional routes, involving a Golgi bypass, are also activated during stress (PSI, RMR1, PS1 and PDF1.2), and the route to the protein storage vacuole seems to be enhanced when compared with the route to the lytic vacuole (upregulation of *VSR1, SYP51* and *VTI12* and downregulation of *VSR2* and *VTI11*). The cytoskeleton adaptor proteins (Net1A) also seem to respond to stress, and a new role for microtubules in cell wall components’ deposition (CesA) may also be important. In the protein storage vacuole, an increase in proteases (like cysteine proteinases) has been documented along with an increase in vacuolar processing enzymes in the lytic vacuole. Ion channels/pumps for the translocation of K^+^, Na^+^ and Mg_2_^+^, located on the tonoplast, are activated in some stress conditions (K/Na pumps and CBL–CIPK), while the blockage of ROS translocation to the vacuole has been observed (RabG3e and VAMP7c). ER—Endoplasmic Reticulum; PSV—Protein Storage Vacuole; LV—Lytic Vacuole; PSI—Plant Specific Insert; ROS—Reactive Oxygen Species; VPEs—Vacuolar Processing Enzymes. Image created with BioRender.com, accessed on 9 January 2022.

The effects of stress in plants have been in the spotlight of research and debated for many decades. However, this discussion has mainly focused on the physiology and the antioxidant system of plants, thereby fundamental processes occurring in cells have often been disregarded. Yet, this is an essential issue to explore, as it has already been established that upon stress a high number of genes and proteins are de novo synthesized and need to be delivered to their proper location. Here, the understanding of the trafficking mechanisms and transport-associated proteins is of utmost importance. Figure 1 represents the changes in the endomembrane trafficking system in cells exposed to adverse environmental conditions and the protein effectors already described as having a role in those processes.

The ER is represented as the main “sensor” for stress and where the stress responses start and from where proteins and signals are either distributed to other locations in the cell or degraded. The vacuole is also extremely relevant in this process, given its multidimensional nature and functions. As such, the vesicular transport between the ER and the vacuole is one of the main processes in plant defence and cellular homeostasis. The high amount of proteins and molecules newly produced will probably cause saturation of the trafficking pathways through the Golgi, as suggested in a recent work where, using Transmission Electron Microscopy [6], it was shown that the Golgi is hypertrophied and associated with high vesiculation in plants under stress. In this scenario, a direct ER-to-vacuole route takes place as a faster route and can be considered as an escape of the traffic jam that originated between the ER, Golgi and prevacuolar compartments. In fact, these unconventional pathways to the vacuole or to the plasma membrane seem to be associated with stress or with other challenging conditions. The mechanisms behind these routes and their regulation are far from being fully understood, but the first steps are being taken and, in the near future, we expect to have a clearer view of the process and a better understanding of the mechanisms behind plant tolerance/adaptation to stress.

## Figures and Tables

**Table 1:** Protein targets involved in abiotic-induced ER stress.

Target	Observations	Reference
BZIP28	Involved in the activation of heat stress response genes	[18,23]
BZIP17	Participates in the activation of salt stress response genes	[20,24]
IRE1	Responsible for the splicing of bZIP60 mRNA, required for the activation of genes involved in the ER stress reaction; regulates the stress transcriptome by degrading several mRNAs	[26,27,28,38]
NPR1	Suppresses the transcriptional role of bZIP28 and bZIP60 in ER stress responses triggered during pathogen attack	[31,32]
ATG8	Following ER stress, many ER components are delivered for degradation via autophagy, forming ER-derived autophagic bodies	[35,36,37]

**Table 2:** Protein targets involved in vacuolar homeostasis in cells under adverse conditions.

Target	Observations	Reference
CBL–CIPK	Important role in the detoxification of Mg2^+^ in the vacuole during salt stress conditions	[60]
VPES	Hydrolytic enzymes, such as proteases and antimicrobial compounds, are released to the cytosolic environment, or extracellularly, to fight pathogen attacks.	[62,63,64]

**Table 3:** Protein targets involved in vacuolar trafficking in cells under adverse conditions.

Target	Observations	Reference
RMR1 VSR1 SYP51 VTI12 VTI11 VSR2	Genes involved in the PSV sorting are positively regulated in plants under abiotic stress, while genes involved in the LV sorting are downregulated	[6]
VSR1	Important for the regulation of abscisic acid (ABA) biosynthesis, a signalling molecule in several stress conditions	[80]
RABG3E	Arabidopsis plants overexpressing AtRabG3e showed increased tolerance to salt and osmotic stress along with a reduction in the accumulation of reactive oxygen species	[81]
VAMP7C	Suppression of the v-SNARE AtVAMP7C had a positive impact in improving plant salt tolerance by inhibiting the fusion of H_2_O_2_-containing vesicles with the vacuole	[83]

**Table 4:** Protein targets associated with unconventional vacuolar routes in cells under abiotic stress.

Target	Observations	Reference
PSIB	Overexpression of PSIB in *Arabidopsis thaliana* correlates with salt and osmotic stress conditions, in some cases improving plant fitness	[89]
CYSTEINE PROTEINASES	Cysteine proteinases accumulate in long ER bodies, whose fusion with the PSV may be triggered by stress	[92]
PR1 PDF1.2	ER bodies filled with defence proteins are formed and eventually fuse with the plasma membrane or with the vacuole in a Golgi-independent manner	[61]

**Table 5:** Protein targets associated with cytoskeleton in cells under stress.

Target	Observations	Reference
CESA	Osmotic stress induces endocytosis of cellulose synthase complex and their interaction with cortical microtubules	[105,106]
CSI1-DEPENDENT SMACCS/MASCS	During endocytosis, CSI1-dependent SmaCCs/MASCs are formed, allowing a quick regulation of cellulose synthesis under abiotic stress	[107]
NET1A	Reacts to extracellular signals, such as stress related to pathogen infection	[108]

## Data Availability

The data presented in this study are available in article.
